# Individual Workplace Well-Being Captured into a Literature- and Stakeholders-Based Causal Loop Diagram

**DOI:** 10.3390/ijerph19158925

**Published:** 2022-07-22

**Authors:** Irene M. W. Niks, Guido A. Veldhuis, Marianne H. J. van Zwieten, Teun Sluijs, Noortje M. Wiezer, Heleen M. Wortelboer

**Affiliations:** 1 Department Work, Health & Technology, The Netherlands Organization for Applied Scientific Research (TNO), 2301 DA Leiden, The Netherlands; marianne.vanzwieten@tno.nl (M.H.J.v.Z.); noortje.wiezer@tno.nl (N.M.W.); 2 Department Defense, Safety & Security, The Netherlands Organization for Applied Scientific Research (TNO), 2509 JG The Hague, The Netherlands; guido.veldhuis@tno.nl; 3 Department Microbiology and Systems Biology, The Netherlands Organization for Applied Scientific Research (TNO), 3700 AJ Zeist, The Netherlands; teun.sluijs@tno.nl (T.S.); heleen.wortelboer@tno.nl (H.M.W.)

**Keywords:** systems thinking, workplace well-being, occupational health, causal loop diagram, group model building, system dynamics, complexity

## Abstract

This study demonstrates an innovative approach to capture the complexity of individual workplace well-being, improving our understanding of multicausal relationships and feedback loops involved. The literature shows that a high number of interacting factors are related to individual workplace well-being. However, many studies focus on subsets of factors, and causal loops are seldomly studied. The aim of the current study was, therefore, to capture individual workplace well-being in a comprehensive conceptual causal loop diagram (CLD). We followed an iterative, qualitative, and transdisciplinary systems-thinking approach including literature search, group model building sessions, retrospective in-depth interviews with employees, and group sessions with human resource professionals, managers, job coaches, and management consultants. The results were discussed with HR and well-being officers of twelve organizations for their critical reflection on the recognizability and potential of the developed CLD. The final result, a conceptual individual workplace well-being CLD, provides a comprehensive overview of multiple, measurable key factors relating to individual workplace well-being and of the way these factors may causally interact over time, either improving or deteriorating workplace well-being. In future studies, the CLD can be translated to a quantitative system dynamics model for simulating workplace well-being scenarios. Ultimately, these simulations could be used to design effective workplace well-being interventions.

## 1. Introduction

In modern societies, workplace well-being is gaining more and more attention. Individual workplace well-being refers to the subjective experience of (a) feeling good (hedonic well-being) and (b) feeling authentic and meaningful in one’s working life (eudaimonic well-being) [1,2]. This experience is not stable but can fluctuate over time. Thus, individual workplace well-being can be conceptualized as a *multidimensional* and *dynamic* concept that characterizes the quality of individual working lives. Employees with a high level of workplace well-being are generally healthier [3], more productive [4] and perform better [5]. However, work-related stress has been significantly increasing over the last few years [6,7,8,9] and has been further aggravated due to the COVID-19 pandemic [10,11]. This indicates that individual workplace well-being is under pressure. In the Netherlands, about 17% of all employees report stress-related complaints, and work-related stress is occupational disease number one [12]. Prolonged work-related stress has negative consequences not only for the health and well-being of workers, but also for the productivity and cost-effectiveness of the organizations they work for [6,13,14]. To combat work stress and maintain or foster employee well-being, effective workplace interventions are needed.

Although employers recognize the benefits of introducing such interventions [15], designing effective organizational well-being promotion programs is still a challenge [16,17,18]. The reason for this challenge lies in the complexity of the concept of workplace well-being. Research shows that a high number of (interrelated) factors can influence workplace well-being, such as job-related factors (e.g., job demands, job resources, and the interpersonal environment), personal resources (e.g., self-efficacy, optimism), and employees’ work–home interfaces (e.g., work–home interference, off-job recovery) [1,19,20]. It is still unknown, however, how all these factors together constitute (different states of) individual workplace well-being. Many studies on workplace well-being focus on small (sub)sets of well-being determinants (see also [21]), and causal loops are seldomly studied. Although empirical research on dynamic interaction mechanisms of well-being is growing [22,23,24], as Sonnentag [1] (p. 285) argues in 2015: “[…] *the underlying processes are probably even more complex than uncovered in past research*”. Intervening *without* a full understanding of individual workplace well-being may result in reduced effectiveness or may even worsen the situation for the individual (see also [25,26,27,28]). For instance, individual coaching aimed at improving employee work-life balance may fail to be effective if a structural imbalance between job demands and job resources prevails, or reducing workload by eliminating tasks that actually provide meaning to one’s work may result in reduced well-being. In summary, for workplace well-being programs to be effective, they should take into account all relevant factors that are at play. To be able to provide guidance to organizations on this matter, a comprehensive view on individual workplace well-being and multicausal and feedback processes through which workplace well-being can be established is necessary (see also review [19]).

The application of a complex systems approach to establish such a comprehensive view on individual workplace well-being has much potential [25,29,30]. Systems thinking offers a methodology to visualize and study the complexity involved [31]. In everyday speech, “complex” often means that “the problem is difficult to tackle”. In complexity science, “complex” refers to a system in which many components are involved that interact with each other in non-linear ways. As a result, a complex system is a whole of which “*the totality is not, as it were, a mere heap, but the whole is something besides the parts*” (Aristotle). The interconnections between system factors can create *feedback loops*, which can be either of a positive (reinforcing) or negative (balancing or goal seeking) nature [30,32,33]. Reinforcing feedback loops tend to amplify change (for instance, the larger a population, the larger the possibility of an increase in birth rate, which further increases the population). Balancing feedback loops, however, counteract change (for instance, the larger a population relative to the carrying capacity of its environment, the lower the net birth rate can be, thereby slowing down population growth [34]). By connecting variables and visualizing causal relation between variables, a so-called causal loop diagram (CLD) can be built. A CLD visualizes how different variables in a system are causally interrelated and which feedback loops are involved. Including factors in the CLD that are *measurable* allows for quantification of the model and simulations of dynamics over time [35]. This methodology can shed light on the consequences of unforeseen interactions based on “what-if” experiments. With respect to individual workplace well-being, such simulations can further enhance insight in the (individual) dynamics of workplace well-being and highlight the possible (or lack of) effectiveness of interventions [36,37]. Ultimately, these new insights could be used to determine which type of workplace well-being intervention would be most effective for whom.

In short, a comprehensive overview of workplace well-being that grasps its dynamic complexity is lacking. We argue that a transdisciplinary systems approach to individual workplace well-being will contribute to filling this gap, by providing a better understanding of multicausal and feedback processes leading to individual workplace well-being. Recently, such an approach has shown promising results for other work-related employee outcomes such as employee performance [29] and the development of burn-out [25], but it has not yet been applied to the topic of workplace well-being. The first step to taking this approach is capturing individual workplace well-being into a CLD. The aim of the present study is therefore to build a conceptual CLD of individual workplace well-being. This CLD will represent a comprehensive view on multiple relevant and measurable factors that together can explain different states and dynamics in individual workplace well-being. In follow-up studies, this CLD can be translated to a quantitative, system dynamics model for designing and simulating the impact of well-being programs within organizations. Based on such simulations, the effectiveness of well-being intervention programs may be improved.

In the next sections, we first describe the multi-methodology and iterative and stepwise approach that we followed to develop the CLD. Next, we present the results with respect to operationalizing individual workplace well-being, key factors relating to workplace well-being, key feedback loops, and the overall conceptual CLD. Finally, we discuss the main results, study strengths and limitations, and future research directions, followed by our main conclusions.

## 2. Methods

### 2.1. Research Team

A research project was set up with three participating institutes, i.e., a project-driven research institute, a health insurance company, and a management consultancy company, all located in the Netherlands. A core research team was in charge of developing the CLD. This team consisted of seven researchers in work and organizational psychology, social psychology, strategy, system dynamics, systems biology and complexity with an experience of 10–30 years in their respective fields of expertise. All researchers are living and working in the Netherlands. The core research team defined the study procedure, as described in the following paragraphs. During the project, results of the (sub steps of the) study were discussed with a stakeholder’s team (team managers, human resource (HR) professionals, job coaches, management consultants) and were reported to a steering committee (higher organizational management) of the three different institutes, who facilitated data collection.

### 2.2. Procedure

The CLD was developed by identifying causal relationships and feedback loops between factors relating to the outcome of the system [34,38], which in our case was defined as individual workplace well-being. To build the CLD, we followed an iterative, qualitative, and transdisciplinary systems thinking approach. Our procedure followed five steps, of which 1–4 iteratively: (1) literature search; (2) defining the boundaries of the system of interest and drafting the CLD; (3) refining the initial version of the CLD with feedback loops based upon retrospective in-depth interviews with employees to capture their perspectives on different states of individual workplace well-being; (4) refining the CLD based upon work sessions with HR professionals, management, job coaches, and management consultants to capture the management perspective; (5) discussing the final CLD by asking HR and well-being officers of twelve client organizations for their critical reflection on the model.

Step 1. Literature search.

A non-systematic, iterative literature search was performed to (a) operationalize individual workplace well-being and (b) identify the key factors related to individual workplace well-being. The focus of the literature search was on original research reports and literature reviews that were published as of the year 2000 in international peer-reviewed journals (focusing on the European work environment). Main search terms that were used were “workplace well-being”, “occupational well-being”, “employee well-being”, and “well-being at work”. We also used the “snowballing” method: going through references of papers already included. The results of the literature search were used to build the CLD in an iterative way. As the research progressed into the model development finetuning phases (see below), we repeatedly searched for additional literature whenever new gaps in the CLD were identified.

Step 2. Defining the boundaries of the system of interest and drafting the CLD.

The boundaries of the system were determined based upon expert knowledge of the core research team, stakeholders’ experience, and the scope of future model application by end users (i.e., employers and employees). This scope was defined as “identifying the relationships of the multiple factors and their feedback loops involved” for “developing, monitoring and evaluating effective well-being promotion programs at the workplace” in the near future. As also mentioned in the introduction, the aim for further research is to quantify the CLD in order to simulate and visualize the effect of possible intervention scenarios on well-being. It was therefore decided to focus only on *measurable* variables. The amount of variables need to be enough to capture the whole complexity of individual workplace well-being but still allowing for future quantification through longitudinal survey studies (without asking too much of participants). In discussion with the core research team and stakeholders, it was decided to focus on the West-European situation. The factors “organizational culture” and “leadership style” were categorized as out of scope, as these variables were presumed to act on a higher, non-individual level, affecting almost all variables in the CLD. During the CLD building process, the core research team closely followed the group model building (GMB) approach as described in detail by Vennix [38]. Each of the sessions lasted 120–180 min. During these sessions, the information gathered from the literature, interviews and reflection work sessions were discussed, gradually building the model based upon consensus. This process was guided by a facilitator and a system dynamics modeler. Discussions during the GMB and collaborative model formulation led to a deep inquiry into the properties of the system, iteratively improving the CLD for the scope defined. VENSIM Simulation Software© developed by Ventana Systems (DSS version 7.0 (Ventana Systems, Inc., Harvard, MA, USA) was used to record and visualize the progress in a causal loop diagram.

Step 3. Refining the CLD with feedback loops based upon retrospective in-depth interviews with employees.

To capture the employee perspective on individual workplace well-being, in-depth interviews with 18 employees from the three participating institutes were held (7, 6, and 5, respectively). Participants were recruited via an email to all employees working in one of the three participating institutes. Inclusion criteria were that a person had gone through a self-perceived period of chronic stress and/or a self-perceived period of chronic work engagement within the last 5 years while working at the same institute. Participants who were interested in participating in this study were invited on a “first come first serve” basis to participate in the study and received an email describing the purpose and outline of the study. The study protocol was in accordance with the World Medical Association Declaration of Helsinki and was approved by the Ethics Committee of TNO.

After signing an informed consent and following a short introduction of the process, participants were first given an assignment to sketch the development of their personal experience of chronic stress and/or work engagement (i.e., period in which the employee was feeling energetic, vital, and motivated at work) over time on a template, following a set of questions (see also [25]). Each of the questions was explained and supported by examples in the questionnaire. Details of the sketch assignments are given in the Supplementary Data. After this task, participants were asked to explain what they had sketched, during an in-depth interview of one hour. Subsequent discussions with the core research team confirmed the key factors and identified dynamic processes of and interactions between key factors involved in individual workplace well-being. These insights were used to refine the CLD.

Step 4. Refining the CLD based upon work sessions with (HR) management.

To capture the perspective of (HR) management on individual workplace well-being, three workshops with delegates from each participating institute were held. Delegates were identified as key informants whose roles within each company directly involved the well-being of employees. Workshop participants were either institute/department manager, operational manager, team manager, HR manager, or supervisor/coach. The workshops took place at each company in a room with audiovisual support and one big table with the CLD printed in A0 format, with the participants sitting around the table. Participants received an overview of the project, the purpose and aims of the workshop, an introduction on the variables in the CLD, and their presumed interactions as an explanation on causal directions. Details of the workshop and assignments are described in the Supplementary Data. During the assignments, participants provided feedback on the CLD by placing sticky notes directly onto the CLD map. The research team recorded the placement and content of notes using photographs and two note takers were present to capture discussions for later analysis. The workshop lasted two hours. Thereafter, the core research team discussed the notes and photographs, and updated the CLD accordingly.

Step 5. Discussing the CLD with HR and well-being officers of twelve client organizations for their critical reflection.

Lastly, the CLD was presented to a group of HR and well-being officers of twelve organizations in the Netherlands, explaining each factor and feedback loop in detail. The group was asked to critically reflect on the CLD and to test the CLD against their own knowledge and experience within their own teams and organization. The feedback of the participants was overlapping in the recognition that no additional determinants and feedback loops could be identified, and that the CLD seemed to capture the whole complexity of individual workplace well-being. Some final adaptations were made in the terminology used.

## 3. Results

First, we briefly present the main findings of the literature search with respect to operationalizing individual workplace well-being. Second, an overview of all identified key factors relating to workplace well-being is presented. Third, the resulting conceptual CLD portraying all variables, causal relations and feedback mechanisms is described. Lastly, an overview of the identified key feedback loops is presented.

### 3.1. Operationalization of Individual Workplace Well-Being

Over the past two decades, a broad range of indicators has been used to operationalize individual workplace well-being. These indicators are either of positive or negative nature. The mostly used *positive* indicators for individual workplace well-being are work engagement and job satisfaction, whereas the mostly used *negative* indicators are burnout complaints, e.g., [39,40,41,42,43]. *Work engagement* is defined as a positive, fulfilling, work-related state of mind, described by the dimensions of vigor, dedication, and absorption [44]. *Job satisfaction* is typically defined as individuals’ global positive feeling about their job [45]. *Burnout complaints* refer to feelings of exhaustion and cynicism toward work, which over time may result in employees taking sick leave and becoming unable to work [46]. We used these three indicators of individual workplace well-being as a starting point for the development of the causal loop diagram.

### 3.2. Key Factors Relating to Individual Workplace Well-Being

Based on the literature findings, employee perspectives (interviews), and management perspectives (workshops), a list of key factors relating to individual workplace well-being was defined. Table 1 provides a list of the key factors in alphabetical order, including a brief description of each factor and examples of literature relevant to the respective factor. The examples of relevant literature are based on a non-systematic, iterative literature search and do not represent an exhaustive list.

### 3.3. Well-Being Model: Causal Loop Diagram

**Figure 1:**
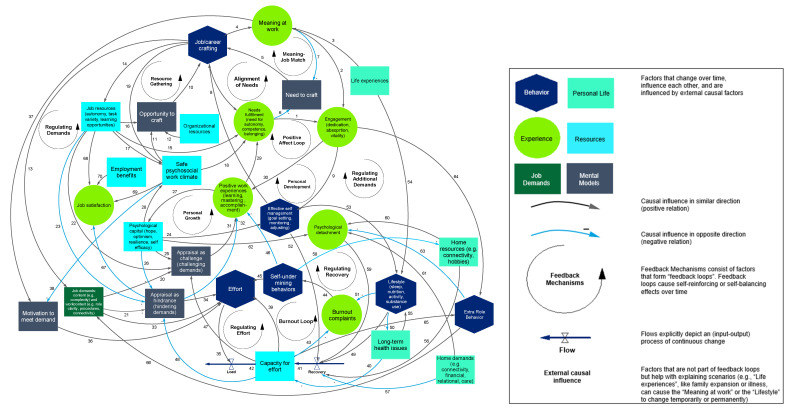
Causal loop diagram of individual workplace well-being. The model consists of several measurable variables classified as aspects of behavior, personal life, experience, resources, job demands, and mental models. Variables are connected with arrows indicating the direction of the causal relationship. Black and blue arrows indicate a positive (reinforcing) and negative (counteracting) causal relationship between variables, respectively. Every causal relationship is numbered to clarify them in the text as *links*. Feedback mechanisms are indicated as circle-arrows in black and are identified with their corresponding names.

Based on the literature and results from the interviews and workshops, the relationships and feedback loops between the key factors relating to individual workplace well-being were defined. Figure 1 depicts the overall resulting CLD of individual workplace well-being. Below, we describe the different parts of the model and the positive (reinforcing) or negative (counteracting) relationships between all factors (referred to as *links*) step by step in more detail.

### 3.4. Work Engagement, Needs Fulfillment, and Meaning at Work

A starting point for the causal loop diagram is work engagement, one of the most widely used positive indicators for individual workplace well-being. Work engagement is characterized by high levels of vigor, dedication, and absorption [44,82]. The literature points at two major predictors of work engagement. The first one is the fulfillment of the basic psychological need for autonomy, competence, and relatedness (*link 1*) [70,71,72]. The need for autonomy is defined as people’s desire to experience ownership of their behavior and to act with a sense of volition [83]. The need for competence represents an individual’s desire to feel capable of mastering the environment, to bring about desired outcomes, and to manage various challenges [84]. Lastly, the need for relatedness is defined as the human striving for close and intimate relationships and the desire to achieve a sense of communion and belongingness [85].

The second major predictor of work engagement is the concept of meaningful work (*link 2*) [67,86,87]. We conceptualized meaning at work as the degree to which someone’s work matches with someone’s personal motives or values (e.g., financial, social, societal) and is experienced as meaningful, valuable and worthwhile. As such, it can entail something else for every individual. Meaning at work can also be subject to change, due to life experiences (*link 3*). Life experiences are emotional events or changes in a person’s life, such as marital status changes, having children, the loss of loved ones, or illnesses. These experiences may cause a shift in personal motives or values. For instance, financial stability may become a stronger personal motive for an employee whose family recently expanded. This shift can result in the need for job/career crafting, which is described below.

### 3.5. Job/Career Crafting and Effective Self-Management

Research shows that an important way to influence the experiences of needs fulfillment and meaning at work is through the proactive behavior of job or career crafting (*links 3 and 4)* (e.g., [60,88]. Job/career crafting can be described as making adjustments to one’s job/career on your own initiative in order to create a better match with one’s personal values, competencies and/or ambitions [59,60,61]. This can be achieved by changing (perceived) characteristics of one’s job/career. For instance, employees may proactively pick up new tasks (i.e., increasing challenging job demands) or ask their supervisor for coaching (i.e., increasing job resources). Crafting can also occur by changing the way of cognitively ascribing meaning and significance to work. An example of this type of crafting is a hospital janitor proactively reframing the purpose of his/her job from “cleaning the hospital” to “contributing to saving people’s lives”.

The degree of job/career crafting, in turn, can be influenced by several factors. First, employees will need to experience a *need* to craft, before engaging in crafting behavior (*link 5*). This need can originate from either a lack of need fulfillment (*link 6*) or a lack of experienced meaning at work (*link 7*). Second, a more general underlying cognitive-behavioral mechanism for job crafting is effective self-management (*link 8*) [53,54]. Effective self-management is about individual goal setting, monitoring one’s progress on reaching these goals, and adjusting goals; basic skills that are necessary for engaging in job/career crafting and that, most likely, are more prevalent when work engagement (i.e., vigor, dedication, and absorption) is high (*link 9*). Third, job/career crafting behavior is influenced by the (perceived) available opportunities to craft (*link 10*) [73,89]. That is, employees may feel either stimulated or restricted to craft their jobs by their direct managers, organizational management, or by cultural factors (e.g., existing behavioral patterns on the job). We expect that both a safe psychological work climate and organizational resources will have a positive influence on perceived opportunities to craft (*links 11 and 12*).

### 3.6. Job Demands, Job Resources and Psychosocial Work Climate

Job characteristics that can be regulated through crafting can generally be divided into job demands (*link 13*) and job resources (*link 14*). Job demands refer to aspects of work that require effort and therefore are associated with certain physiological and psychological costs [46]. Examples are emotional demands, complexity, role ambiguity, and multitasking. Job resources, however, refer to aspects of work that (a) are functional in achieving work goals, (b) reduce job demands, or (c) stimulate personal growth and development. Examples of job resources are job autonomy, performance feedback, task variation, and development opportunities.

Research shows that job resources are important predictors of positive well-being outcomes [71,73]. With regard to processes through which this happens, job resources have been directly related to the fulfillment of basic needs (*link 15*) [71]. In addition, job resources such as autonomy and development opportunities will most likely contribute to employee (perceived) opportunities to craft their jobs (*link 16*) [73]. Further, the availability of job resources such as feedback and instrumental support may drive positive social interactions, which are part of a safe psychosocial work climate (*link 17*). Key elements of a safe psychosocial work climate are positive social interactions based on trust and respect, supportive leadership processes, and social support [1,80]. In a safe psychosocial work climate, employee well-being is a priority. As a consequence, employees feel psychologically safe, supported, and free to raise problems and difficult topics. This most likely translates to a higher sense of belonging (needs fulfilment; *link 18*). There is also a positive relation between job crafting and a safe psychosocial work climate, as social support from supervisors and colleagues can be crafted (*link 19*) [60].

With respect to job demands, the literature shows that the effect of job demands on well-being outcomes is highly dependent of the way they are appraised by the individual [51,52]. That is, demands can be appraised as potentially promoting personal growth and achievement (i.e., challenge stressors; *link 20*) or as potentially constraining their personal development and work-related accomplishment (i.e., hindrance stressors; *link 21*). For instance, employees may perceive a complex assignment as an opportunity to learn new things, or as an extra workload that may hinder them from achieving other tasks. In general, challenge demands can be related to positive well-being outcomes, whereas hindrance demands can be related to negative well-being outcomes [52]. The way demands are appraised by the individual will (partly) depend on the availability of job resources in the work environment (*links 22 and 23*), as job resources can help employees to effectively deal with demands [46]. Another important factor that contributes to the appraisal of job demands is the concept of psychological capital, which we discuss next.

### 3.7. Psychological Capital and Positive Work Experiences

Psychological capital (PsyCap) refers to an individual’s positive psychological state of development, characterized by self-efficacy, optimism, hope, and resiliency [77]. Employees in possession of these psychological capacities are more likely to engage in effective self-management (link 24) and to appraise job demands as challenges rather than hindrances (*links 25 and 26)* [90]. PsyCap is open to development through interventions (e.g., training, coaching) or through positive work experiences (*link 27*) [76]. Examples of such positive experiences are receiving recognition (e.g., compliments, rewards) and mastery (i.e., learning experiences including “small wins”). That is, through recognition or learning experiences, employees can form stronger positive evaluations about themselves. In turn, employees with high PsyCap capacities actively show positive behaviors [91], which contributes to a safe psychosocial work climate (*link 28*).

Positive work experiences do not only contribute to PsyCap, but also to a higher needs’ fulfillment (*link 29*), for instance by increasing ones’ sense of competence (through mastery) or belonging (through receiving recognition). We expect that positive work experiences are more likely to occur when work engagement is high (*link 30*) and when job demands are appraised as challenges rather than hindrances (*links 31 and 32*).

### 3.8. Effort and Motivation to Meet Demands

Meeting job demands inevitably requires a certain degree of effort from employees (*link 33*) [49]. The intensity of the required effort depends on the quantity and nature of the specific job demands one is dealing with. Dealing with hindrance demands will most likely require additional effort (*link 34*), as those demands are perceived as an “obstacle” rather than as a positive challenge. The higher the degree of effort one puts into his or her work, the higher one’s experienced workload will be (*link 35*).

Effort expenditure also depends on individual motivation to meet demands (*link 36*), which is based on his or her expectations of the consequences of (not) meeting those demands. For instance, someone that is afraid of negative results when not completing a task (e.g., unhappy customer or colleague, negative performance appraisal) may go above and beyond to complete the task after all. Motivation to meet demands, in turn, is influenced by two factors. First, it is likely that employees who experience their work as highly meaningful, valuable and worthwhile (meaning at work) will also have a high motivation to meet demands (*link 37*). Second, we expect that in a safe psychosocial work climate, employees will experience less fear of negative consequences of *not* meeting demands, resulting in a lower motivation to meet demands against all costs (*link 38*).

Lastly, the extent to which employees put effort into work will depend on their individual capacity to do so (*link 39*). This concept is discussed in the next paragraph.

### 3.9. Capacity for Effort and Burnout Complaints

Capacity for effort refers to an individuals’ overall physical and mental capacity for putting effort into work, which may be impacted by long-term health issues (*link 40*) (e.g., physical disabilities, chronic diseases). Aside from such health issues, capacity for effort results from the (im)balance between workload and recovery (*links 41 and 42*) [49]. That is, when recovery from work is insufficient, one’s capacity for effort will decrease. Over time, this may result in burnout complaints (*link 43*). Burnout complaints are a commonly used negative indicator of workplace well-being and can be characterized by feelings of exhaustion and cynicism toward work [46]. Employees suffering from such negative feelings are more likely to engage in behavior that undermines their job performance, such as making (more) mistakes, communicating poorly, or getting into personal conflicts (*link 44*) [81]. By having to make up for these self-undermining behaviors, their effort increases (*link 45*), which can further decrease their capacity for effort. In addition, self-undermining behavior will most likely be negatively related to positive work experiences (*link 46*).

Capacity for effort can also be linked to the “personal development loop”, through the appraisal of job demands (*links 47 and 48*). More specifically, we expect that employees with a *high* capacity for effort will be more likely to appraise job demands as a challenge rather than a hindrance, and the other way around.

### 3.10. Recovery and the Work-Home Interface

Burnout complaints and the recovery aspect of capacity for effort are subject to various factors in the work–home interface. First, a healthy lifestyle (i.e., sufficient sleep and exercise, healthy diet, no substance abuse) is inducive to recovery from work (*link 49*), can prevent certain long-term health issues (*link 50;* e.g., type 2 diabetes), and can serve as a buffer against the development of burnout complaints (*link 51*). Both recovery and lifestyle itself can be positively influenced by effective self-management *(links 52 and 53)*. Lifestyle can also be subject to change, for better or for worse, due to life events (*link 54*; e.g., becoming a parent) or long-term health issues (*link 55;* e.g., physical disabilities or concentration problems due to brain injury).

Second, recovery can be respectively facilitated or hampered by home resources and demands (*links 56 and 57*). Home resources can be conceptualized as aspects in one’s personal (nonwork) life that give energy (e.g., spending time on hobbies, positive social interactions). Home demands refer to those aspects in one’s personal life that drain energy (e.g., relational conflicts, financial worries). Home demands may be present for a number of reasons beyond the scope of the model but can also be a result of self-undermining behavior (*link 58*; e.g., taking work frustrations out on family or friends).

Third, an important predictor for recovery is the concept of psychological detachment from work (*link 59)* [78,79]. It refers to the degree to which individuals mentally and emotionally “switch off” of work and are no longer engaged in work-related matters during free time. Factors that can positively contribute to detachment from work are home resources and demands (*links 60 and 61*; i.e., aspects that require taking one’s mind off work) and psychological capital (*link 62*; i.e., possessing the psychological capacity to detach from work). A factor that can negatively affect detachment from work is extra-role behavior (*link 63*).

### 3.11. Extra-Role Behavior

Extra-role behavior is defined as work-related behavior that is not officially part of the job description and is therefore not necessarily expected of someone, but that is useful and contributes to organizational goals (e.g., deepening in specific content, learning new skills, commitment to staff association) [58]. This type of behavior is often described as “going the extra mile” and is positively influenced by work engagement (*link 64*) [92,93]. Another predictor of extra-role behavior is capacity for effort: a higher capacity for effort makes it more likely for employees to go above and beyond (*link 65*). Although this behavior may seem quite desirable from the organizational perspective, there is a potential downside: employees may use part of their free time to engage in this type of behavior, which prevents individuals from taking one’s mind off of work (*link 63*). In addition, taking on extra tasks means an increase in job demands (*link 66*). Consequently, required effort increases and the balance between workload and recovery may be jeopardized.

### 3.12. Job Satisfaction

Lastly, we end the causal loop diagram with job satisfaction. Job satisfaction refers to an individuals’ global positive feeling about their job, consisting of an extrinsic and intrinsic component [65]. The intrinsic component is about how people feel about the nature of the work itself, which can be influenced by their appraisal of job demands (*link 67*), the availability of job resources (*link 68*), and the psychosocial work climate (*link 69*) [94,95]. The extrinsic component is about how people feel about external aspects of the work situation, such as salary and benefits. This component will clearly be influenced by the conditions of employment offered by the employer (*link 70*). Similar to work engagement, job satisfaction is a commonly used positive indicator of workplace well-being. However, a clear difference with work engagement in this causal loop diagram is that for job satisfaction, there are no outgoing relationships to other well-being-associated factors. That is, job satisfaction does not seem to elicit further positive gain spirals and, as such, may not be the best target outcome for workplace well-being interventions.

### 3.13. Key Feedback Loops Underlying Workplace Well-Being

Table 2 provides a list of key feedback loops in alphabetical order, including the type of the feedback loop (reinforcing or balancing), the key variables involved, and a brief description of each feedback loop. This list is not exhaustive; additional feedback loops can be found in the model. However, the feedback loops presented in Table 2 are most central to the CLD and together cover most of the key variables in the CLD.

## 4. Discussion

The literature shows that individual workplace well-being can be conceptualized as a dynamic, multidimensional concept in which a high number of factors related to individuals’ mental and physical states as well as to the context are involved. These factors may interact over time. However, many studies focus on specific parts of workplace well-being, whereas multicausality and feedback loops are seldom studied. We argued that a dynamic systems approach to individual workplace well-being might help unravel multicausality and feedback loops. In turn, this may provide new insights that can be used to design effective organizational workplace well-being promotion programs. In dynamic systems approaches, causal loop diagrams (CLDs) are used for mapping the multicausality of complex and dynamic issues, including bio-psychosocial issues within the health domain [25,36,96,97]. The aim of the current study was, therefore, to take a first step toward a systems approach by developing a conceptual causal loop diagram of individual workplace well-being. To do so, we integrated scientific literature and qualitative data on perspectives on workplace well-being from well-being experts, employees, and (HR) management.

The developed conceptual CLD provides a comprehensive overview of multiple key factors relating to individual workplace well-being and of the way these factors may interact over time, either improving or deteriorating individual workplace well-being. By focusing on *measurable* factors, future studies can aim to quantify the model through longitudinal data collection and analyses. In this step, it will be crucial to collect the appropriate data describing the whole system [98]. Such data allow for the presumed relations in the CLD to be verified and enable simulations of dynamics over time [35]. Simulations of individual workplace well-being could, for example, focus on questions such as: to what extent and within what time frame would burnout complaints of an individual most likely diminish when there is a positive change in their psychological capital? What is likely to happen to work engagement when psychological detachment improves? Would that process be different for employees with a low versus a high motivation to meet demands? Such insights may be used to select and/or develop suitable interventions for specific scenarios (e.g., training or coaching trajectory aimed at increasing psychological capital). Simulations of individual workplace well-being scenarios could, in turn, highlight the potential effects of workplace well-being interventions over time [36,37]. More in-depth information about this approach can be found in recent literature. For instance, a proof of concept was recently shown for burnout on an individual level [25]. In this study, Veldhuis and colleagues successfully simulated differential behavior patterns in the development of burn-out and recovery for three personas (i.e., fictitious persons with pre-determined scores on a set of key variables in the model). Similarly, Alefari et al. [29] developed a quantitative system dynamics model of employee performance, illustrated by a case study with three virtual experiments. Another recent study demonstrated how dynamic simulations can be used to design lifestyle intervention programs based on scenarios tested through “what-if” experiments [36]. Scenario simulations of possible effects of intervention policies have been illustrated by others before in other domains [98]. Ultimately, new insights brought about by this approach could be used to design effective organizational workplace well-being promotion programs.

### 4.1. Strengths and Limitations

A strength of the current study is the transdisciplinary and systems approach. To the best of our knowledge, this is the first study that applies dynamic systems thinking to workplace well-being. Although the development of the causal loop diagram can be seen as merely a first step to taking a dynamic systems approach, it opens the way to future research directions that can be of added value to both theory and practice, as also described above. In particular, the model can be used to determine appropriate questionnaires and organizational variables for monitoring (the progress of) individual workplace well-being and, as such, contribute to determining the effectiveness of well-being intervention programs (with pre–post measurement studies).

Because of the high number of drivers of individual workplace well-being, another strength of the current study is the integration of different existing theoretical models and concepts, existing empirical evidence, and newly collected perspectives from both employees and (HR) managers in one causal loop diagram. The non-systematic, iterative literature search helped to (a) operationalize individual workplace well-being and (b) identify the key factors and their interactions related to individual workplace well-being. This provided a successful starting point for collaboratively developing the CLD together with the well-being of scientists, employees and professionals in the field. The addition of employee experiences of chronic stress or work engagement based upon retrospective in-depth interviews with employees contributed to the unraveling of causal relationships. These interviews did not only focus on the (static) key variables, but also and especially on the dynamic relations between variables over time. The evaluation of the model with various (HR) managers improved the model even further, by thoroughly verifying that the model captured the whole complexity of individual workplace well-being into one CLD. As a result, a number of important feedback loops were identified.

A third strength is that the CLD focuses on both individual and contextual aspects of workplace well-being. As such, the model may support personal transformations to a “better version of the individual”, contextual transformations to a “better environment for well-being”, or combinations between these two.

Besides strengths, some limitations can also be identified. First, a CLD of a complex issue as individual workplace well-being can never be 100% complete. Although the CLD integrates a large body of existing literature, the literature search was non-systematic, and some studies may have been omitted. In addition, the current CLD is based on a limited amount of personal data. Then again, the model was thoroughly and iteratively evaluated, and consensus between all participating stakeholders was reached that no substantial factor seemed to be missing from the final version of the model. Second, the participants (employees, managers) were all highly educated and working at organizations located in the Netherlands. The model may therefore not be (fully) generalizable to other groups of employees. Lastly, as discussed earlier, it was decided not to incorporate organizational culture and leadership style as separate variables into the model. However, it is expected that these factors differ highly between organizations and may have a major influence on many variables and relationships involved in the model. Future studies are needed to further investigate this issue. Meanwhile, it is important not to disregard these factors when assessing individual workplace well-being.

### 4.2. Future Research

Further research is needed to evaluate and calibrate the model. Because we focused on measurable factors in building the CLD, we expect that future studies can use methods available in computational modeling to quantify the CLD. Our research consortium has the ambition to do so. The first step would be to quantify the CLD into a system dynamic model with a first set of longitudinal workplace well-being data. To involve different sectors, a well-being community of organizations has already been formed in the Netherlands. With a participative role of the organizations, employees, and well-being researchers, we aim to collect data of the effectiveness of workplace well-being programs and the processes involved at the level of the individual. Such data will give us the opportunity to identify personal dynamic patterns and to further improve the model. Ultimately, the quantified model could be used to determine which type of well-being intervention would be most effective and for whom.

## 5. Conclusions

This study demonstrates the feasibility of systems thinking and a transdisciplinary innovative approach to capture the complexity of individual workplace well-being, improving our understanding of the multicausal relationships and feedback loops involved. We conceptualized different states of individual workplace well-being as the outcome of complex and dynamic interactions between various variables in so-called feedback loops. As such, this work is complementary to scientific approaches focusing on workplace well-being and the efficacy of intervention programs that do not take such dynamics into account. The next step will be the translation of the conceptual CLD into a quantitative system dynamics model, followed by calibration and validation of the model using empirical data of longitudinal participatory action studies. As such, the model may become a (research) tool to design well-being promotion programs and support individual employees, teams, and (organizational) managers in their decisions to improve individual workplace well-being.

## Figures and Tables

**Table 1:** Key factors relating to workplace well-being, description of key factors and examples of relevant literature.

Key Factors	Description	Literature
Burnout complaints	Feelings of exhaustion and cynicism toward work	[46,47,48]
Capacity for effort (load vs. recovery)	An individual’s physical and mental capacity for making efforts, resulting from the (im)balance between workload and recovery	[49]
Challenge demands (appraisal as challenge)	Job demands (see: job demands) that are positively assessed as challenging because they lead to the development of new knowledge or skills and the achievement of work goals (e.g., complex assignment, new role)	[50,51,52]
Effective self-management	Self-regulatory behavior in which an individual (a) sets individual goals, (b) monitors the achievement of these goals and (c) adjusts goals and/or adjusts behavior when necessary	[53,54]
Effort	Degree of effort an individual puts in at work	[49,55]
Employment benefits	Terms of employment such as working hours and salary	[56,57]
Extra-role behavior	Work-related behavior that is not officially part of the job description and is therefore not necessarily expected of someone but that is useful and contributes to organizational goals (e.g., deepening in specific content, learning new skills, commitment to staff association)	[58]
Hindrance demands (appraisal as hindrance)	Job demands (see: job demands) that are negatively assessed as obstructing, because they are in the way of achieving work goals (e.g., conflict, role ambiguity, interruptions, organizational politics)	[50,51,52]
Home resources	Aspects of someone’s private life that energize someone (e.g., hobbies, social interactions).	-
Home demands	Aspects of someone’s private life that cost energy (e.g., relational conflicts, financial worries).	-
Job/career crafting	Proactively making small adjustments to one’s job/career in order to create a better match with one’s personal values, competencies and/or ambitions. This can be achieved by increasing challenging demands, reducing hindering demands, increasing (social) job resources, or changing the way of cognitively ascribing meaning and significance to work.	[59,60,61]
Job demands	Job demands are those work characteristics that demand energy and/or effort from the employee (e.g., emotional demands, complexity, role ambiguity, multitasking).	[46,62,63]
Job resources	Job resources are those work characteristics that can help employees to achieve work goals, deal with job demands, and learn new things (e.g., job autonomy, feedback, task variation, development opportunities).	[46,62,63]
Job satisfaction	Individuals’ global positive feeling about their job, consisting of an extrinsic and intrinsic component. The intrinsic component is about how people feel about the nature of the work itself. The extrinsic component is about how people feel about external aspects of the work situation, such as salary and benefits.	[45,64,65]
Life experiences	Emotional events or changes in a person’s life, such as marital status changes, becoming a parent, illness, illness or loss of loved ones, career changes	-
Lifestyle	Lifestyle factors such as sleep, diet, exercise, substance abuse	-
Long-term health issues	Health complaints that are chronic or long-lasting	-
Meaning at work	Degree to which someone’s work matches with someone’s personal motives or values (e.g., financial, social) and is experienced as meaningful, valuable and worthwhile	[66,67]
Motivation to meet demands	Motivation to meet job demands, based on an individual’s expectations of the consequences of (not) meeting those demands	[68]
Need to craft	Need for job/career-crafting behavior, arising from the degree of experienced (mis)fit between current and desired work situation	[69]
Needs fulfilment	Degree to which the three basic psychological needs (i.e., competence, relatedness and autonomy) are fulfilled	[70,71,72]
Opportunity to craft	Extent to which an employee experiences opportunities for job/career-crafting behavior within the organization	[73]
Organizational resources	Organizational-level job resources that can help employees to achieve work goals, deal with job demands, develop, and learn new things (e.g., HR practices)	[46]
Positive work experiences	Positive experiences at work (e.g., mastery, receiving positive feedback)	[74,75]
Psychological capital (PsyCap)	A set of personal resources at work: self-efficacy, optimism, hope and resilience.	[76,77]
Psychological detachment	Degree to which individuals mentally/emotionally “switch off” of work and are no longer engaged in work-related matters during free time	[78,79]
Safe psychosocial work climate	Positive interpersonal work environment in which employees feel safe, feel free to raise problems and difficult topics and experience social support.	[1,80]
Self-undermining behaviors	Behavior that creates obstacles that undermine one’s job performance (e.g., making (more) mistakes, poor communication, engaging in personal conflicts).	[81]
Work engagement	A positive, fulfilling, work-related state of mind, described by the dimensions of vigor, dedication, and absorption	[44,82]

**Table 2:** Key feedback loops underlying workplace well-being, type, key variables involved, and short description.

Name Key Feedback Loop	Type Reinforcing /Balancing	Key Variables Involved	Short Description
Alignment of needs	balancing	Job/career crafting Need fulfillment Need to craft	When individuals’ needs are more fulfilled (due to more job/career crafting, positive work experiences, safe psychological climate or job resources), there is a possibility that the need to craft will reduce, reducing job/career crafting and therefore reducing needs fulfillment.
“Burn-out” loop	reinforcing	Burnout complaints Self-undermining behavior Effort Capacity for effort (load vs. recovery)	The more burnout complaints, the higher the possibility that self-undermining behavior increases. To make up for individuals’ self-undermining behavior, more effort is needed, and workload increases. This further decreases their capacity for effort which, in turn, reinforces burn-out-complaints.
Meaning-job match	reinforcing	Meaning at work Need to craft Job/career crafting	When the degree to which someone’s work matches with someone’s personal motives or values (e.g., due to a life experience), there is a possibility that the need to craft will increase, enhancing job/career crafting, improving the meaning at work.
Personal development	reinforcing	Effective self-management Job/career crafting Meaning at work Engagement	More effective self-management increases the possibility that an individual may engage in a higher level of job/career crafting, improving individuals’ meaning at work and engagement, and reinforcing effective self-management
Personal growth	reinforcing	Positive work experiences Psychological capital (PsyCap) Challenging demands (appraisal as challenge)	The more positive work experiences, the larger the possibility that the psychological capital of the individual is enhanced, which improves the assessment of job demands as challenging, as they lead to the development of new knowledge or skills and the achievement of work goals, which leads to positive work experiences
Positive affect loop	reinforcing	Work engagement Needs fulfillment Positive work experiences	The more work engagement (more vigor, dedication, and absorption), the larger the possibility that an individual has more positive work experiences, improving needs fulfillment, which leads to more work engagement
Regulating demands	balancing	Job/career crafting Job demands Challenging demands (appraisal as challenge) Hindering demands (appraisal as hindrance) Positive work experiences Needs fulfillment Need to craft	Through job/career crafting, employees can increase the level of job demands that they appraise as challenging and/or reduce demands that they appraise as hindering, leading to more positive experiences at work and needs fulfillment, which in the end reduces the need to craft enhancing job/career crafting
Regulating Effort	balancing	Effort Capacity for effort (load vs. recovery)	Putting more effort into work will reduce once’s capacity for effort (increased load). As a result, the level of effort will decrease.
Regulating extra demands	balancing	Engagement Extra role behavior Psychological detachment Capacity for effort (load vs. recovery) Challenge demands Work experiences Need Fulfillment	More engagement may enhance extra role behavior, increasing job demands and reducing psychological detachment, thereby reducing recovery and capacity for effort, reducing challenge demands, reducing positive work experiences, reducing needs fulfillment, in the end reducing engagement
Regulating recovery	reinforcing	Psychological detachment Capacity for effort (load vs. recovery) Effort Challenge demands (appraisal as challenge) Positive work experiences	The more psychological detachment, the larger the possibility of improving recovery, improving capacity for effort, increasing the appraisal of demands as challenging and decreasing the appraisal of certain demands as hindering, increasing positive work experiences, psychological capital, and psychological detachment
Resource gathering	reinforcing	Job/career crafting Job resources Opportunity to craft	Job/career crafting behavior can increase the level of available job resources and can enhance the opportunity to craft, which can stimulate further job/career-crafting behavior

## Supplementary Materials

The following are available online at https://www.mdpi.com/article/10.3390/ijerph19158925/s1, Supplementary S1: Supplementary Data. Reference [25] is cited in the supplementary materials.
