# Supplementary material for: Individual Workplace Well-Being Captured into a Literature- and Stakeholders-Based Causal Loop Diagram
Source: Int J Environ Res Public Health. 2022 Jul 22;19(15):8925. doi: 10.3390/ijerph19158925 (PMC9331132; doi:10.3390/ijerph19158925)
Supplement: Supplementary file 1 [file ijerph-19-08925-s001.zip › ijerph-1798360-supplementary.pdf]

Supplementary Data

Collection of Retrospective longitudinal data by sketching graphs-over-time

To capture the employee perspective on individual workplace well-being, a group of fifteen employees were asked to provide retrospective longitudinal data by sketching graphs-over-time, as described earlier[1]

In short, in-depth interviews by a researcher from the core research team with each participant were held. After a short introduction of the process, participants were first given an assignment to sketch the development of either chronic stress or engagement over time on a template, following five questions. Each of the questions was explained and supported by examples in the questionnaire. Identify the start and end date of your chronic stress or engagement experience on the x-axis and include the following assignments in the timeline.

a. Identify developments in your professional life and mark during which time they were active.
b. Identify developments in your personal life and mark during which time they were active;
c. Sketch graphs of the following 7 variables as perceived by yourself during the timeframe: engagement, stress-responses, amount of (job) demands, job resources, interpersonal work environment, meaning at work, and psychological capital.
d. Identify developments in personal job crafting and mark during which time were active
e. Reflect on your personal graph and actions and mark during which time you could have conducted interventions to improve the situation

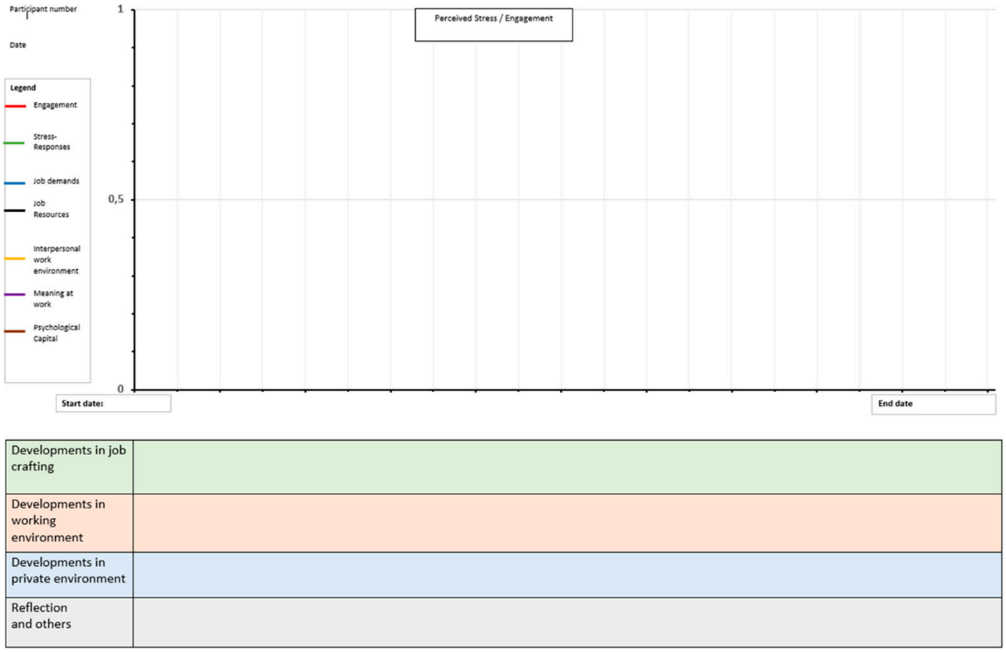

Figure S1. Template sketch assignment, translated from Dutch to English

[1] Veldhuis, Guido A., Teun Sluijs, Marianne H. J. van Zwieten, Jildau Bouwman, Noortje M. Wiezer, and Heleen M. Wortelboer. 2020. "A Proof-of-Concept System Dynamics Simulation Model of the Development of Burnout and Recovery Using Retrospective Case Data." *International Journal of Environmental Research and Public Health* 17(16):1–28

Fifteen participants were asked to "quantify" and to "draw their own dynamics" for seven variables (i.e., engagement, stress reactions, load, resources, interpersonal working environment, meaning at work, psychological capital) in relation to four timelines (job crafting, developments in the work environment, developments in personal environment, reflection and others). All participants gave their informed consent for inclusion before they participated in the study, and to use their personal experience and insights for incorporation in the model. All fifteen participants completed the sketching task in 60 min. A typical sketch (digitalized with Microsoft PowerPoint) is presented in Figure 2 and two examples of response are presented in Figure 3.

a

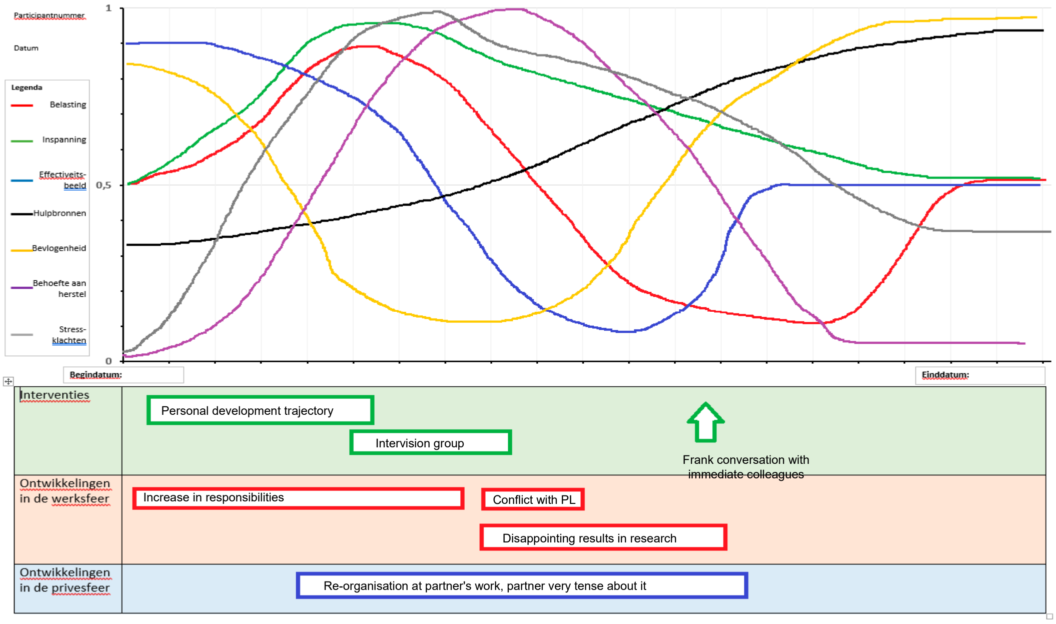

b

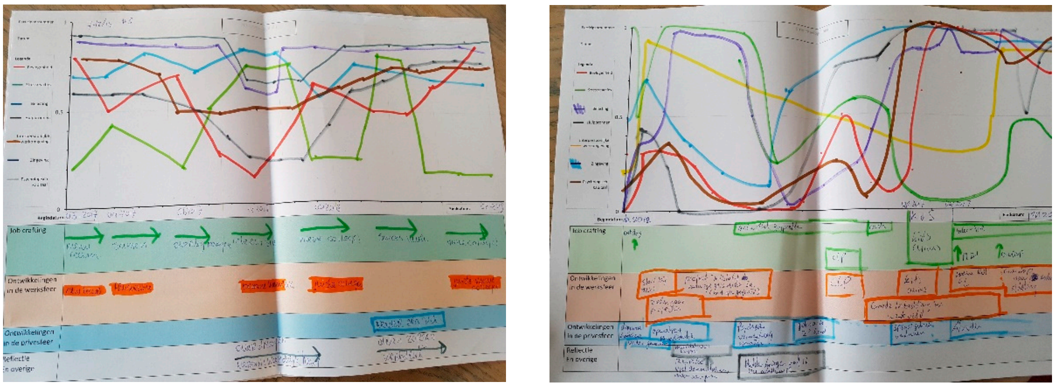

Figure S2. The response from one of the participants digitalized with Microsoft PowerPoint (a) and two pictures of the actual responses of two participants (b). The top section shows the graphs for seven perceived variables (i.e., engagement, stress reactions, load, resources, interpersonal working environment, meaning at work, psychological capital) sketched by the participants. The bottom section shows the four timelines (from-top-to-bottom: job crafting, developments in the work environment, developments in personal environment, reflection and others. The actual responses included notes on the developments as can be seen in the two pictures below.

Refining the CLD based upon work sessions with (HR)department, management, coaches

To capture the perspective of (HR)department, management, coaches and management consultants on individual workplace well-being, evaluate and refine the CLD even further, three workshops with delegates from Human Resource, job coaching, and management per each participating.

Participants were asked to share their input on the CLD based on the following three questions:

a. If you think of employees becoming more engaged, do you recognize the current variables, causal relations and feedback loops? Do you miss any variables? Do you agree with the relative importance of various factors?
b. If you think of employees becoming less engaged, do you recognize the current variables, causal relations and feedback loops? Do you miss any variables? Do you agree with the relative importance of various factors?
c. What can HR/coaching/management do to influence various variables and processes? (looking ahead, discuss practical model applications: directions for wellbeing programs/interventions)

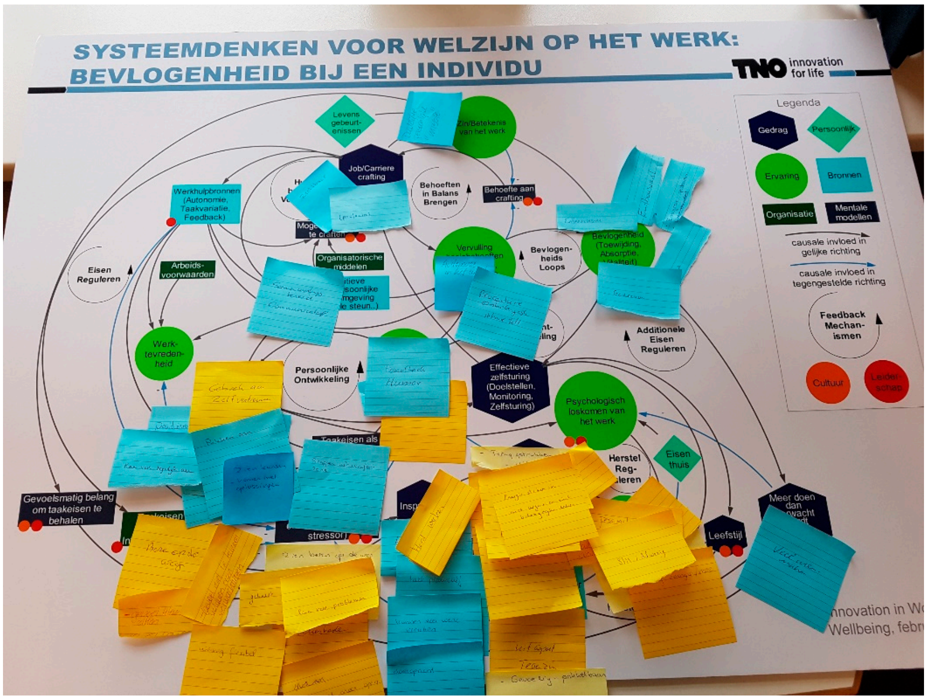

Figure S3. Typical result of a workshop with delegates from Human Resource, job coaching, and management.
